# Regulated Transport into the Nucleus of Herpesviridae DNA Replication Core Proteins

**DOI:** 10.3390/v5092210

**Published:** 2013-09-16

**Authors:** Alvisi Gualtiero, David A. Jans, Daria Camozzi, Simone Avanzi, Arianna Loregian, Alessandro Ripalti, Giorgio Palù

**Affiliations:** 1 Department of Molecular Medicine, University of Padua, Padua 35121, Italy; E-Mails: simone.avanzi@unipd.it (S.A.); arianna.loregian@unipd.it (A.L.); 2 Department of Biochemistry and Molecular Biology, Monash University, Victoria 3800, Australia; E-Mail: david.jans@monash.au; 3 National Research Council of Italy, Institute of Molecular Genetics, Unit of Bologna, Bologna 40136, Italy; E-Mail: daria.camozzi@gmail.com; 4 Azienda Ospedaliera Universitaria di Bologna Policlinico S. Orsola-Malpighi, Bologna 40138, Italy; E-Mail: alessandro.ripalti@unibo.it

**Keywords:** herpesviruses, replicase, CK2, inhibitors, anti-viral therapy, nuclear transport regulation, importins, phosphorylation, hsp90

## Abstract

The *Herpesvirdae* family comprises several major human pathogens belonging to three distinct subfamilies. Their double stranded DNA genome is replicated in the nuclei of infected cells by a number of host and viral products. Among the latter the viral replication complex, whose activity is strictly required for viral replication, is composed of six different polypeptides, including a two-subunit DNA polymerase holoenzyme, a trimeric primase/helicase complex and a single stranded DNA binding protein. The study of herpesviral DNA replication machinery is extremely important, both because it provides an excellent model to understand processes related to eukaryotic DNA replication and it has important implications for the development of highly needed antiviral agents. Even though all known herpesviruses utilize very similar mechanisms for amplification of their genomes, the nuclear import of the replication complex components appears to be a heterogeneous and highly regulated process to ensure the correct spatiotemporal localization of each protein. The nuclear transport process of these enzymes is controlled by three mechanisms, typifying the main processes through which protein nuclear import is generally regulated in eukaryotic cells. These include cargo post-translational modification-based recognition by the intracellular transporters, piggy-back events allowing coordinated nuclear import of multimeric holoenzymes, and chaperone-assisted nuclear import of specific subunits. In this review we summarize these mechanisms and discuss potential implications for the development of antiviral compounds aimed at inhibiting the Herpesvirus life cycle by targeting nuclear import of the Herpesvirus DNA replicating enzymes.

## 1. Introduction

The *Herpesviridae* family comprises several human pathogens of pivotal clinical importance, including the α-*Herpesvirinae* herpes simplex virus type 1 and 2 (HSV-1/-2) and Varicella Zoster virus (VZV), the β-*Herpesvirinae* human cytomegalovirus (HCMV) and human herpesvirus 6 and 7 (HHV-6/-7), as well as the γ-*Herpesvirinae* Epstein-Barr virus (EBV) and Kaposi’s sarcoma-associated herpesvirus (KSHV), (see [1] for a review). Although differing in important properties such as host specificity, clinical manifestations, and *in vitro* growth kinetics, all *Herpesviridae* family members replicate their linear double stranded (ds) DNA genome in the nucleus of productively infected cells. Lytic viral DNA replication is a process that starts at defined sites on the viral genomes, the origin of DNA replication (ori_Lyt_). Most β-herpesviruses have a single ori_Lyt_, whereas several α- and γ-herpesviruses have two (VZV, EBV and KSHV) or three (HSV1, HSV2), respectively. Although all herpesviruses control replication initiation at ori_Lyt_ via virus-encoded DNA binding polypeptides, a *bona fide* ori-binding protein (OBP) has been identified only in α-herpesviruses and some β-herpesviruses, while in HCMV and γ-herpesviruses this function is performed by viral proteins also transactivating viral gene expression. The exact molecular events that lead to herpetic DNA replication are still mysterious, with several lines of more recent evidence putting into question the dogma of a rolling circle-based replication, instead supporting a recombination-dependent branching mechanism [2]. Despite this, there is general agreement regarding the formation of concatamers during the process, from which genomic units need to be resolved before viral genome encapsidation occurs. 

Regardless of its exact mechanism, viral DNA replication takes place within the host cell nucleus and is mediated by a number of cellular and viral proteins [3,4,5,6,7]. The latter include an OBP, a DNA polymerase holoenzyme, composed by a DNA-dependent DNA polymerase catalytic subunit (pol) and a DNA polymerase accessory protein (PAP) conferring processivity to pol, as well as a trimeric helicase/primase complex formed by a primase, an helicase and a primase associated factor (PAF). Finally, a single stranded (ss) DNA-binding protein is also required. These factors sequentially assemble on the ori_Lyt_ and mediate viral genome replication. Therefore, herpesviral replication fork proteins need to reach the nucleus after having been translated in the cytoplasm. Here we summarize current knowledge regarding signals and transporters responsible for nuclear targeting of herpesviral DNA replication proteins and the mechanisms regulating this process, with a focus on possible strategies to impair viral replication by interfering with it.

## 2. Nuclear Import

A hallmark of eukaryotic cells is the high degree of compartmentalization. The genetic information resides in the cell nucleus, separated from the cytoplasmic apparatus responsible for protein synthesis by a double membrane structure, the nuclear envelope (NE). The passage of molecules such as mRNA and proteins through the NE occurs necessarily through aqueous channels delimited by huge NE-embedded multiprotein-complexes called nuclear pore complexes (NPCs) [8]. It is generally accepted that molecules smaller than 50 kDa can freely diffuse through the NPCs [9], while the transport of larger molecules is an active, signal-dependent process. Among these molecules, cellular mRNAs and pre-miRNAs need to be exported from the nucleus after transcription to the cytoplasm in order to be translated or processed by DICER, respectively, while proteins such as histones and lamins, need to be imported into the nucleus after having been synthesized in the cytoplasm to reach the DNA or the inner NE, respectively [10,11].

**Figure 1:**
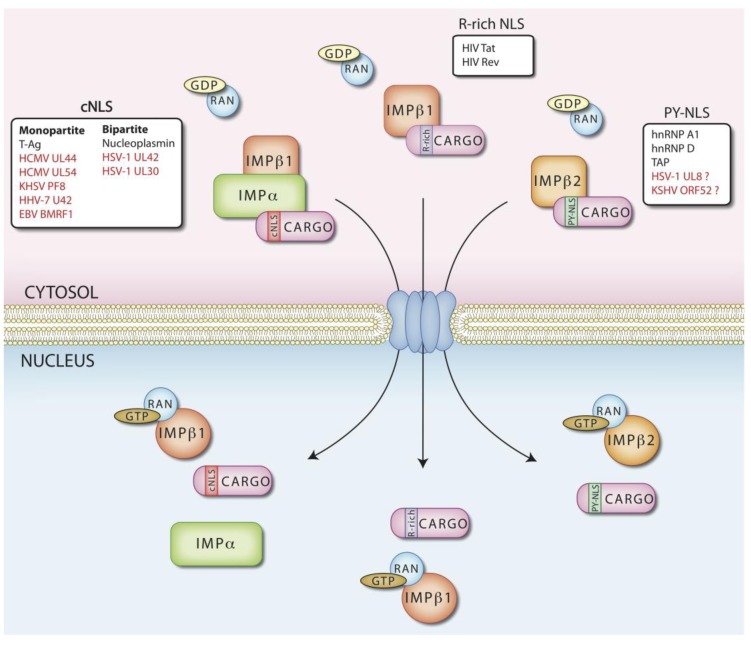
Cellular transporters involved in signal dependent nuclear targeting of herpesviral proteins. Transport of cargoes across the nuclear envelope (NE) occurs exclusively through channels delimited by the nuclear pore complexes (NPCs), upon recognition of specific nuclear localization complexes (NLSs) by specialized cellular transporters, with different signals being recognized by a variety of transporters: the IMPα/β1 heterodimer recognizes cNLSs, which can either be of the monopartite or bipartite type (A), importin IMPβ1 recognizes arg-rich NLSs (B), whereas IMPβ2 recognizes PY‑NLSs. Proteins carrying the various types of NLS are listed in the boxes, with prototypical NLSs being shown in black, and herpesviral DNA replicase NLSs indicated in red.

Active transport of molecules across the NPC-delimited channel is a signal-mediated process effected by transporters belonging to the importin (IMP) β superfamily of cellular transporters that recognize specific targeting signals on cargoes. These signals can be divided into nuclear localization sequences (NLSs) and nuclear export sequences (NESs), responsible for targeting into and out of the nucleus, respectively (Figure 1) [12]. The study of viral proteins generally has proven to be invaluable in delineating the key molecular details of many eukaryotic cell processes [13,14], with nucleocytoplasmic transport being no exception. The first evidence of nuclear targeting signals stemmed from the work of Kalderon *et al*. with respect to simian virus 40 (SV40), that showed that SV40 large tumor antigen (T-ag), a protein normally localizing in the host cell nucleus, mislocalized to the cytoplasm upon mutation of a short stretch of basic amino acids (aas) [15]. Importantly, fusion of such a sequence to otherwise cytoplasmic proteins was sufficient to confer nuclear localization [16]. The 7-aas highly basic sequence P**KKKRK**V (single letter aa code) was delineated as an NLS, sufficient and necessary to confer T-ag nuclear localization [15,16]. Since then, a number of different types of NLS have been identified which can be classified on the basis of their sequence, and their ability to be recognized by different IMPs, and NLSs similar to that of T-ag are often referred to as “classical” NLSs (cNLSs) [17], which are highly basic, generally lysine-rich, and confer direct interaction with the adapter protein IMPα complexed to IMPβ1 (see below). cNLSs can be classified as monopartite or bipartite, according to the number of basic stretches of aas composing them. Monopartite NLSs resemble the T-ag-NLS and are a single cluster of basic residues, matching the consensus (K-K/R-X-K/R), while the bipartite NLSs, with two clusters of basic residues separated by a spacer region of 10–13 aa, resemble the NLS from the *Xenopus laevis* histone assembly factor nucleoplasmin (**KR**-11 aas-**KKKK**) [17]. IMPα is formed by two distinct domains: a short, basic *N*-terminal IMPβ-binding (IBB) domain responsible for binding to IMPβ1, and the remainder of the molecule comprising 10 Armadillo (ARM) tandem repeats, including two regions of ARM repeats representing the major and minor NLS-binding sites [18]. In the absence of IMPβ1, the IBB domain competes with the NLSs for the major NLS-binding site, resulting in an auto-inhibitory mechanism [19]; IMPα-IMPβ interaction enables NLS-binding by displacing the IBB domain. Monopartite NLSs bind preferentially to the major NLS binding site, while bipartite NLSs interact with both binding pockets. The complex is subsequently docked to the NPC and translocates through it as mediated by IMPβ, before reaching the nucleus. Once inside the nucleus, binding to IMPβ of the small Ras-related guanine nucleotide binding protein Ran in activated GTP-bound form, results in dissociation of IMPβ from the IBB domain of IMPα, which in turn competes for the major NLS binding site on IMPα, thus effecting cargo release into the nucleus [20] (Figure 1). Importantly, the conformation of two key structural loops in Ran depends on the nucleotide bound, which thereby determines the affinity for IMPβ homologues, regulating the interaction between the latter and the cargoes [21,22]. The RanGTP/RanGDP ratio is low in the cytoplasm and high in the nucleus, ultimately ensuring directionality of the nuclear transport process [23]. This RanGTP gradient is maintained by the asymmetrical distribution of two key regulators of Ran nucleotide state: Ran activating GTPase (RanGAP), which is predominantly found in the cytoplasm [24], and the nuclear Ran guanine nucleotide-exchange factor (RanGEF, also known as RCC1), which mediates substitution of GDP with GTP [25]. Cytoplasmic RanGDP is imported into the nucleus by its specific transporter nuclear transport factor 2 (NTF2) [26], and subsequently converted to RanGTP by the action of RCC1. As mentioned above, IMPα/IMPβ1-cargo complexes are dissociated in the nucleus after interaction of IMPβ with RanGTP [20]. The IMPβ-RanGTP complex is subsequently recycled to the cytoplasm, while IMPα is exported from the nucleus by the IMPβ homologue CAS, as complexed with RanGTP [27]. Once in the cytoplasm, the action of RanGAP catalyzes the hydrolysis of GTP to GDP, resulting in the dissociation of RanGDP from the IMPs which are then available for a new round of nuclear import, while the newly generated RanGDP can be transported into the nucleus by NTF2 [26].

As mentioned above, cargoes can be imported into the nucleus through several possible pathways, in addition to the classical nuclear import pathway, whereby the IMPα/β heterodimer mediates import of a cNLS bearing cargo. Seven different IMPα homologues have been identified in humans, and can be assigned to three subgroups, on the basis of homology and cargo binding specificity [28]. Alternatively, IMPβ1 can recognize the m3G-cap of snRNAs via the adaptor Snurportin 1, to mediate nuclear import of snRNPs [29]. Additionally certain basic NLSs are recognized specifically by IMPβ1 directly [30,31,32]; an example is the **R**QA**RR**N**RRRR**W**R** sequence from the human immunodeficiency virus (HIV-1) Rev protein [33]. Finally, certain atypical NLSs, such as the one recently described on KHSV LANA, can also be directly recognized by IMPβ1 [34].

On the other hand, the almost 20 IMPβ1 homologues described so far bind cargoes directly without the need for an adaptor such as IMPα [35,36]. Of these, IMPβ2 is the best characterized, with > 20 cargoes, mostly RNA binding proteins, already identified, including the RNA binding protein heterogeneous ribonucleoprotein particle A1 (hnRNP-A1), which contains the 30 aa M9-NLS (FGNYNNQSSNFGPMKGGNFGG**R**SSGPYGGG) [37]. IMPβ2 recognized NLSs have been identified in various other RNA binding proteins [38,39,40,41], but crystallographic and biochemical approaches were necessary to enable definition of the predictive consensus PY-NLS, consisting of highly disordered sequences of about 20–30 residues, which can be divided in *N*- and *C*-terminal motifs. The consensus sequence determined for the latter (R/K/H-X(2,5)-PY) is highly conserved in the majority of identified PY-NLSs, wherein substitution of key aas impairs recognition by IMPβ2 [42,43]. In contrast, the *N*-terminal motif has a looser consensus, formed by either a hydrophobic or basic stretch of aas, the bPY-NLS and hPY-NLS subclasses respectively. The basis of this appears to be the existence of two NLS binding sites embedded inside the *C*-terminal domain of IMPβ2: the “A” site, necessary for strong and specific interaction with the R/K/H-X(2,5)-PY motif and the “B” site, which is rich in acidic residues but with hydrophobic residues scattered throughout, enabling either basic or hydrophobic linear peptides to be accommodated. Mutational analysis on either basic or hydrophobic aas within PY-NLSs results in reduced interaction with IMPβ2, confirming the requirement of mutated residues for proper interaction. Thus, it is likely that the role of the A site is essential, with the B site being required to increase IMPβ2 binding affinity [43,44]. Besides differences in target protein recognition, IMPβ2 and IMPβ1 share the same mechanisms for cargo transport and release into the nuclear compartment. In the case of IMPβ2, cargo release is through Ran-GTP binding to a disordered loop of IMPβ2 (H8 Loop), which contributes to the B site, facilitating cargo release from IMPβ2 into the nuclear compartment [43,44].

## 3. Regulation of Nuclear Transport

Regulation of protein transport across the NPC, and the kinetics thereof, plays a central role in controlling cellular processes such as cell growth and differentiation, and response to pathogens [45]. In the case of transcription factors, this can determine whether a critical intranuclear concentration is achieved or not, thus ultimately determining its effect on cellular transcription [46]. Mechanisms regulating nuclear import include alteration of the expression levels and/or function of IMPs, or components of the NPC [47], and/or modulation of the affinity of IMP recognition of the cargo by post-translational modification, such as by phosphorylation [46]. The latter strategy offers several advantages, including the ability of modulating intranuclear protein concentration without requiring *ex-novo* protein synthesis or degradation, and affords rapid and reversible control of nuclear/cytoplasmic localization [48]. Indeed the localization of several crucial cellular key molecules is controlled by this mechanism, including the Drosophila morphogen Dorsal, where protein kinase A (PKA) mediated phosphorylation 22 residues upstream of the NLS are associated with a c. seven-fold increase in IMPα binding affinity, and a marked increase in transport efficiency [49]. Another extremely well characterized example of phosphorylation-regulated nuclear import of cargo is the SV40 T-ag, where two synergistically acting kinases (protein kinase CK2 and dsDNA-dependent kinase) phosphorylate residues located 10–12 aa upstream of the NLS, thus increasing the affinity of NLS recognition by IMPα/β1 by about 100-fold to achieve high efficiency of T-ag nuclear targeting [50]. T-ag nuclear import is also controlled by a phosphorylation site for the cyclin-dependent kinase cdk1 adjacent to the NLS, where phosphorylation confers binding to the cytoplasmic retention factor BRCA-1 binding protein 2 (BRAP2), a general negative regulator of nuclear import [51].

## 4. Nuclear Transport of Herpesviral DNA Replicating Enzymes

In order to mediate viral DNA replication, the proteins forming the herpesviral DNA replication core need to reach the cell nucleus. Besides ssDNA binding protein, the two main complexes are a multimeric DNA polymerase holoenzyme and helicase/primase complexes. Given the high molecular weight of such proteins, their nuclear transport has been shown to be an active process mediated by a number of different IMPs, which recognize specific NLSs on the cargo proteins [52,53,54,55,56,57,58,59]. In some cases, individual subunits are capable of localizing to the cell nucleus independently of the others, like in the case of HSV and HCMV DNA polymerase holoenzyme subunits [52,53,54,55,56]. However in the case of KSHV and EBV DNA polymerase holoenzymes [59,60] and most of their helicase/primase complex subunits, formation of the holoenzyme appears to be a pre-requisite for nuclear transport [61,62,63,64] (see Table 1).

**Table 1:** Identified NLSs and nuclear import pathways of Herpesviral DNA replicating enzymes. The NLSs identified so far for each herpesviral DNA replication proteins are indicated, as well as the IMPs binding them. The subcellular localization of each protein when individually expressed is also indicated (loc. alone). Additional details are reported in respect to the nuclear import pathways of DNA polymerase and helicase/primase holoenzymes. Pol, DNA polymerase catalytic subunit; PAP, DNA polymerase processivity factor: hNLS, hydrophobic NLS; cNLS, classical NLS; bip, bipartite NLS; N, nucleus; C, cytoplasm; IMPs, importins.

	HSV-1				HCMV			
*protein*	*name*	*NLS*	*IMPs*	*loc. alone*	*name*	*NLS*	*IMPs*	*loc. alone*
processivity factor	UL42	1 cNLS bip [55]	a/β	N [55]	UL44	1 cNLS [52]	a/β	N [52]
DNA polymerase	UL30	1 cNLS bip [53] 1 NLS hyd [53,56]	a/β	N [53,56,87]	UL54	1 cNLS [54] 1 NLS hyd [56]	a/β	N [56]
primase	UL5			C [63,64]	UL70			C [52]
helicase	UL52			C [63,64]	UL105			N in infected cells [110]
primase-associated factor	UL8	putative PY-NLS (see text for details)		C [63,64]	UL102			
ssDNA binding protein	UL29			?	UL57			
DNA polymerase holoenzyme nuclear import	Individual subunits and holoenzyme [55]				Individual subunits and holoenzyme [54]			
PAP status	Monomer [76]				Dimer [77,106]; transported to the nucleus as dimer [105]			
Helicase primase complex nuclear import	Transported as a trimeric complex C [63,64]				Not known, but UL70 can localize to the nucleus independently of the other subunits if the cellular chaperone DNAJB6-a is overexpressed [109]			
	**EBV**				**KHSV**			
*protein*	*name*	*NLS*	*IMPs*	*loc. alone*	*name*	*NLS*	*IMPs*	*loc. alone*
processivity factor	BMRF1	1 cNLS [84]		N [84]	ORF59	1 cNLS [59]		N [59]
DNA polymerase	BALF5			C [60]	ORF9			C [59]
primase	BSLF1			C [61]	ORF44	putative PY-NLS (see text for details)		C [59]
helicase	BBLF4			C [61]	ORF56			C [59]
primase-associated factor	BBLF2/3			C [61]	ORF40/41			C [59]
ssDNA binding protein	BALF2				ORF6			
DNA polymerase holoenzyme nuclear import	Piggy back as mediated by PAP [60]				Piggy back as mediated by PAP [59]			
PAP status	Multimer [78,114]				Dimer [79,115 ]; transported to the nucleus as dimer [116]			
Helicase primase complex nuclear import	Imported as a complex; BBLF4 can be imported if expressed with the ZTA transactivator [ 61]				pol/PAP and all the helicase primase subunits need to be simultaneously expressed to achieve nuclear targeting [62]			

## 5. DNA Polymerase Holoenzyme Nuclear Transport

**Figure 2:**
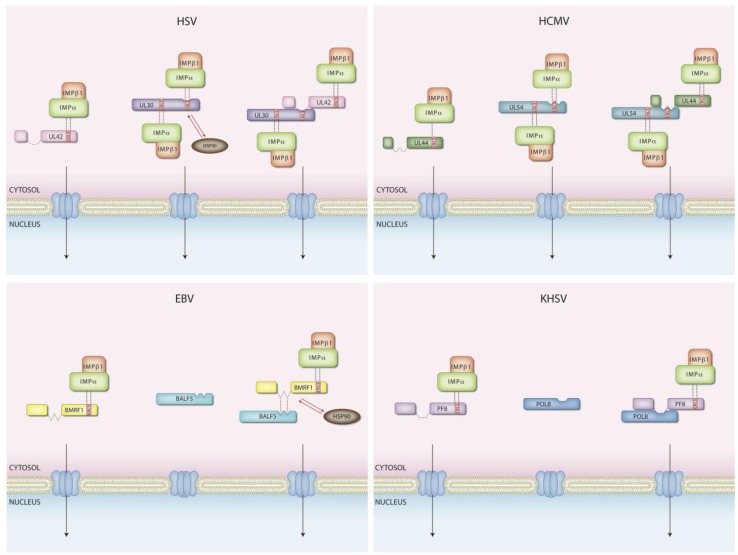
Nuclear transport of DNA polymerase holoenzymes. Different herpesviruses have evolved distinct mechanisms to mediate nuclear targeting of their DNA polymerase holoenzymes. In the case of HSV-1 and HCMV both pol and PAP enzymes possess intrinsic, NLSs and can be imported to the nucleus either alone or as a fully assembled enzyme. In contrast, EBV and KSHV pols are incapable of nuclear targeting in the absence of the respective PAPs, bearing a *C*-terminally located NLS. A requirement of Hsp90 activity has also been reported for the nuclear import of HSV-1 and EBV pol. Black vertical bars indicate direct protein-protein interactions; red vertical bars indicate direct protein-protein interactions depending on Hsp90 activity.

All human herpesviruses encode a two subunit DNA polymerase. The pol subunit is known to perform the synthesis of leading and lagging strands, while the PAP tethers the holoenzyme to the DNA, thus conferring processivity [65,66,67]. Since the replication of all known herpesviruses takes place in the host cell nucleus [68], these high molecular weight proteins need to be actively transported through the NPC to reach the site of viral DNA replication. Different herpesviruses have evolved different strategies to ensure correct subcellular localization of their DNA polymerase holoenzyme. Indeed different proteins can be translocated independently, or only as fully assembled holoenzymes, wherein a NLS present on the PAP mediates import of the catalytic subunit via a piggy-back mechanism. In all known herpesviruses, a functional NLS has been characterized on the PAP [52,55,57,58,59], which plays a central role in nuclear targeting of the holoenzyme (Figure 2 and Table 2). In addition, in the case of HCMV a complex phosphorylation-dependent regulation system finely tunes nuclear import of the PAP [51,52,69,70]. Herpesviral PAPs can be generally divided into two functional domains: a *N*-terminal catalytic domain and a *C*-terminal nuclear targeting domain. The *N*-terminal domain is capable of performing all known biochemical activities, including binding to pol and to dsDNA in the absence of ATP and clamp loaders, and stimulation of the holoenzyme activity [71,72,73,74,75]. Structural studies showed that the *N*-terminal domains of HSV-1 (UL42), HCMV (UL44), EBV (BMRF1) and KSHV (PF-8) PAPs share a very similar three dimensional architecture, which closely resembles that of monomers of the eukaryotic DNA polymerase processivity factor PCNA, the main difference being that UL42 is a monomer, while UL44, BMRF1 and PF/8 form head to head dimers [76,77,78,79,80]. On the other hand, the *C*-terminal domains appear to be largely unstructured, do not possess any known biochemical property, and carry an NLS. Functional NLSs have been identified at the *C*-terminus of HSV-1, HCMV, HHV-7, EBV and KSHV PAPs, all of which are known to be able to localize independently of pol into the cell nucleus [52,55,57,58,59], probably reflecting the fact that they are also capable of performing additional functions during the viral life cycle. The latter hypothesis is also suggested by several other lines of evidence, including their higher and more temporally delayed expression kinetics when compared to pol, and the fact that they have been shown to possess transcriptional regulation properties [81,82,83,84,85]. In the case of HCMV, HHV-7, EBV and KSHV PAPs, the cNLS is monopartite, closely resembling that identified on SV40 T-ag [52,57,58,59]. In the case of HSV-1, the NLS is also located at the *C*-terminus, but appears to be bipartite, with two basic clusters of aas being required for optimal nuclear targeting [55]. Biochemical and functional studies revealed that nuclear import of such cargoes is energy and Ran-dependent, and mediated by the IMPα/β1 heterodimer [55,57] (see Table 2). It is not clear why HSV-1 has evolved a bipartite NLS on its PAP UL42, but this might reflect the ability of HSV-1 to infect neuronal cells *in vivo*. Indeed different IMPα members exhibit unique temporal expression profiles in neurons [86], and immunoprecipitation experiments have shown that UL42’s bipartite NLS can bind to both IMPα-S and α-Q members, while the HCMV PAP UL44 monopartite NLS only binds efficiently to IMPα-S members [55]. Regardless of these differences, all known PAPs bear a functional NLS. In contrast, not all known pol subunits are able to localize to the cell nucleus independently of their PAP. Indeed, while HSV-1 and HCMV pols [87] or portions thereof [54] localize to the cell nucleus when expressed in the absence of other viral proteins in cell culture, this is not the case for pols from EBV and KSHV, wherein NLSs have not been identified, and nuclear targeting entirely relies on the presence of the respective PAP via a piggy-back mechanism [59,60]. The reason why these viruses evolved such different strategies to ensure nuclear targeting of their DNA polymerase catalytic subunits is not clear, but it is possible that the ability of HSV and HCMV DNA pols to be independently transported into the nucleus is critical to their infectious cycles. Indeed, both viruses evolved two distinct but functional NLSs on their pol enzymes. The first to be discovered were atypical hydrophobic NLSs (hNLSs), which lie within the PAP binding domain, and thus appear to be active only when pol is not associated with the PAP [56]; these have not been fully characterized. cNLSs, at the very *C*-terminus of the proteins outside the PAP binding domains similar to those identified in the respective PAPs (see below) were subsequently described that confer energy- and Ran-dependent nuclear targeting to reporter proteins, as well as recognition by the IMPα/β heterodimer through direct interaction with IMPα [53,54]. In addition, in contrast to the hNLSs, which are masked upon PAP binding [54,55], these signals are functional in the context of the DNA polymerase holoenzymes. Due to the presence of these additional NLSs, HSV and HCMV pols are capable of translocating their respective PAPs to the nucleus via a piggy-back mechanism once the NLS on the latter are inactivated by mutagenesis [54,55]. Thus, it appears that HCMV and HSV have evolved several mechanisms to maximize the nuclear import possibilities of their DNA polymerase subunits, so that they are capable of both being imported individually or as complexes in holoenzymes. In contrast, functional NLSs are not present on the pols from EBV and KSHV-8 and their nuclear translocation entirely relies on their interaction with their PAP. Although the IMPs responsible for nuclear targeting of the EBV and KSHV-1 PAPs have not been identified experimentally, the process is likely mediated by the IMPα/β heterodimer, since EBV and KSHV PAPs possess highly basic sequences resembling the T-ag cNLS (see Table 2). This apparently obligates the cotranslocation into the nucleus of DNA polymerase holoenzyme subunits in the case of KSHV and EBV, and likely provides a means for coordinating and facilitating the processes of viral DNA recognition and processive DNA synthesis.

The fact that several herpesviral DNA polymerase subunits are imported by the IMPα/β heterodimer has important implications for antiviral therapy. Indeed Ivermectin, a broad-spectrum anti-parasite medication has been recently identified as a specific inhibitor of IMPα/β-mediated nuclear import [88]. Treatment of cells expressing proteins translocated to the nucleus by the IMPα/β heterodimer such as HCMV UL44 and 54, as well as Dengue virus NS5 and HIV-1 integrase (IN), resulted in a strong inhibition of nuclear localization of such cargoes [89]. More importantly, treatment of cells infected with HIV-1 and DENV resulted in a strong reduction of virus production [89,90]. It is therefore not unreasonable to suggest that inhibition of IMPα/β nuclear transport could also have a broad anti herpetic activity. 

**Table 2:** NLSs identified on Herpesviral DNA polymerase subunits and nuclear import molecular mechanisms. Pol, DNA polymerase catalytic subunit; PAP, DNA polymerase processivity factor; hNLS, hydrophobic NLS; cNLS, classical NLS. The single letter amino acid code is used; hydrophobic residues in hNLS are *underlined*, whereas basic residues in cNLSs are in *boldface*. s indicates the ability of the NLS to function when the subunit is uncomplexed; h indicates the ability of the signal to function once the holoenzyme is assembled. In the column “Ran” the sensitivity of nuclear transport to overexpression of the transdominant negative RanQ69L is reported; in the column “ATP”, sensitivity to depletion of intracellular ATP levels; X, sensitivity; ?, unknown.

Virus	Protein	Name	NLS type	Sequence	IMPs	Ran	ATP	Nuclear import	Notes	Reference
HSV-1	pol	UL30	hNLS	RRMLHR-1129	?	?	?	s	overlapping PAP binding domain	[56]
			cNLS bip	PA**KR**P**R**ETPSPADPPGGAS**K**P**RK**-1136	a/β	X	X	s + h		[53]
	PAP	UL42	cNLS bip	PTT**KR**G**R**SGGEDARADAL**KK**P**K**-413	a/β	X	X	s + h		[55]
HCMV	pol	UL54	hNLS	PRRLHL	?	?	?	s	overlapping PAP binding domain	[56]
			cNLS	PA**KKR**A**R**-1159	a/β	X	X	s + h		[54]
	PAP	UL44	cNLS	PNT**KK**Q**K**-431	a/β	X	X	s + h	regulated by phosphorylation	[52]
HHV-7	pol	U38	/	/	/	/	/	/		
	PAP	U27	cNLS	PNS**KR**Q**R**-361	?	X	X	s		[57]
KHSV-1	pol	pol-8	no NLS					h	requires PF-8 for import	[59]
	PAP	PF-8	cNLS	**KR**PH**KRR**SD-377	?	?	?	s + h	mediates translocation of holoenzyme	[59]
EBV	pol	BALF1	no NLS					h	requires BMRF1 for import	[60]
	PAP	BMRF1	cNLS	**K**HP**KK**-396	?	?	?	s + h	mediates translocation of holoenzyme	[58]

## 6. MCV UL44 by IMPs

**Figure 3:**
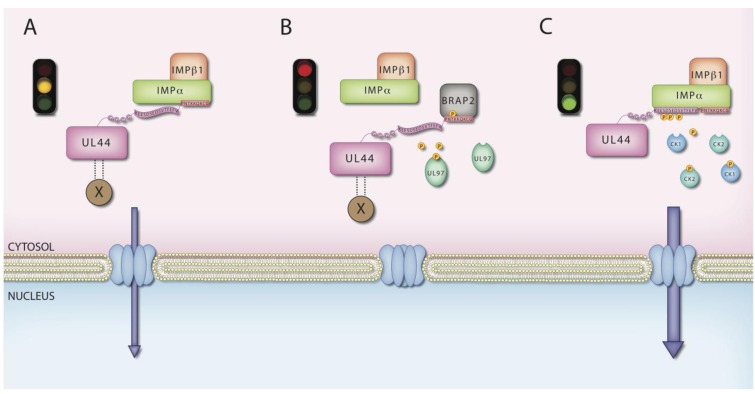
Phosphorylation-regulated nuclear transport of HCMV PAP. HMCV PAP UL44 nuclear import depends on the phosphorylation of its *C*-terminus. (**A**) Unphosphorylated UL44 *C*-terminus can be recognized by the IMPα/β heterodimer with medium affinity. This results in a low rate nuclear import of UL44, possibly enabling interaction with other cytoplasmic factors before import occurs. X indicates either another UL44 molecule, UL54 or the viral uracil DNA glycosylase; (**B**) Phosphorylation of T427, as mediated by UL97 or cdk1 enables binding to the cytoplasmic retention factor BRAP2, thereby preventing IMPα/β recognition and nuclear targeting. Phosphorylation of S413, S415 and S418 by CK2 and CK1 enhances IMPα/β binding, thereby promoting rapid nuclear import, possibly preventing UL44 to interact with other partners before nuclear import (**C**).

Beside inhibition of the IMPα/β dependent nuclear import generally, with associated problems of host cell toxicity, a potentially more specific approach to inhibit viral replication would be to inhibit the recognition between specific NLS bearing viral cargoes and the transporter [91], as recently described for the HIV-1 matrix protein [92] and for the HCMV viral phosphoprotein UL84, which plays a crucial, still not yet completely characterized role during viral infection [93]. Indeed, the above mentioned redundancy of NLS on DNA polymerase holoenzymes of HSV-1 and HCMV but not on EBV and KSHV implies that the NLSs located on the PAPs from the latter represent potential targets for the development of antiviral drugs, since their function in not redundant for viral replication in cell culture [59,60]. On the other hand, in the case of both HSV and HCMV, simply targeting the PAPs NLSs might not be sufficient to block viral replication, since inhibition of DNA polymerase holoenzymes nuclear transport would require the simultaneous inhibition of the cNLSs located on the PAPs and on the catalytic subunits. Consistent with this hypothesis, HSV-1 UL42 *C*-terminal domain, containing the functional cNLS and absolutely required for nuclear import of UL42 when expressed individually [55], is completely dispensable for virus growth in cell culture [94]. Surprisingly, this is in stark contrast to reports for HCMV, where deletion of the HCMV UL44 *C*-terminal domain, including the cNLS [52], results in the inability of HCMV to replicate in cell culture [95,96]. This is surprising, given the fact that the UL44 *C*-terminal deletion mutant can still localize to the nucleus through piggy-back by the UL54 pol catalytic subunit, and addition of the T-ag cNLS does not restore viral replication [52,95,96]. The clear implication is that the UL44 *C*-terminal domain plays other roles in the HCMV life cycle, in addition to conferring nuclear localization to the protein. The UL44 *C*-terminus (residues 409–433) is in fact the target of extensive post-translational modifications. These include multiple phosphorylation sites (S413, S415, S418 and T42T) and a sumoylation site (K410), which regulate UL44 subcellular localization by controlling its ability to interact with host cell proteins [52,69,70,97,98], although the exact role of UL44 sumoylation is still unknown [97]. Depending on the target residues, phosphorylation can either negatively or positively regulate UL44 nuclear import. Indeed, *in vitro* data showed that the viral kinase UL97 is capable of phosphorylating T427. This residue lies within UL44 cNLS sequence (PNT**KK**Q**K**431) and mediates interaction with the cellular cytoplasmic retention factor BRAP2 [51,99]: negative charge on T427 enables BRPA2 binding and reduces UL44 nuclear accumulation more than 10-fold [51,69]. Thus, phosphorylation of T427 can prevent UL44 interaction with the IMPα/β heterodimer by two mechanisms: first of all, the negative charge in position +1 of the NLS can directly decrease binding to the IMPα NLS-binding pocket due to electrostatic repulsion between the phosphate group of the T residue and the NLS binding groove [70,100]. Secondly, binding of BRAP2 to UL44 competes directly with IMPα/β for binding to the NLS [51,69]. In contrast, phosphorylation of S413, S415 and S418 strongly increases UL44 nuclear accumulation in transient transfection assays [52]. Intriguingly, the three residues are part of a phosphorylation cascade triggered by protein kinase CK2 (CK2) that phosphorylates S413, that in turn promotes protein kinase CK1 (CK1) phosphorylation of S418, which then enables CK2 to phosphorylate S415 [69]. Analysis of UL44 subcellular localization of UL44 point mutant derivatives demonstrates that each phosphorylation event increases UL44 nuclear accumulation [69], with pharmacological inhibition of CK2 able to reduce nuclear accumulation [52,70]. The molecular basis for improved nuclear import through phosphorylation of S413, S415 and S418 appears to reside in altered binding affinity for the IMPα/β heterodimer, in similar fashion to SV40 T-ag and the key role of the CK2 site upstream of its NLS [50]. Consistent with this idea, a GFP-UL44(410–433) fusion protein containing sequences upstream of the NLS binds to IMPα/β with much stronger affinity than a GFP-UL44(425–433) fusion protein containing only the NLS, as shown using native gel electrophoresis [52]. Strikingly, the key phosphorylation sites in HCMV UL44 are conserved in the HHV-6 and seven homologues of UL44, suggesting that β-herpesviruses utilize a common strategy to enhance nuclear import of their PAPs [52]. These regulatory mechanisms, which enable the extent of nuclear targeting of a cargo to be controlled by modulating the affinity of recognition between the IMPs and the cNLS within the cargo itself, are analogous to those originally described for the SV40 T-ag [101]. In the latter case, CK2- and dsDNA-dependent kinase cooperatively phosphorylate T-ag upstream of its NLS to enhance its nuclear localization and cdk1 phosphorylates in close proximity to the NLS itself to prevent nuclear targeting; comparable regulatory mechanisms apply to other viral (Human papillomavirus 11-E1; see [102]) and cellular proteins (for example p53; see [48]). HCMV has thus evolved to be able to finely tune the nuclear transport of its PAP by modulating its interaction with the specific IMPs responsible for PAP nuclear targeting. Therefore UL44 can be translocated to the nucleus at three different rates (Figure 3), according to its phosphorylation status, which is of importance in the context of the viral life cycle. Indeed, when UL44 is phosphorylated on residues S413, S415 and S418, its NLS is recognized with very high affinity by IMPα/β, resulting in highly efficient/rapid nuclear accumulation. This may prevent UL44 from associating with other viral and cellular proteins in the cytoplasm, thus supplying a source of uncomplexed nuclear protein able to perform DNA replication-related (for example by associating with UL54) or unrelated (for example by regulating transcription [83]) functions. Similarly, phosphorylation of T427 by UL97 or cdk1/2 [69,103] would prevent interaction with IMPα/β by promoting interaction with BRAP2, causing UL44 to be retained in the cytoplasm, to perform other as yet unknown functions. Finally, a lack of phosphorylation on UL44 *C*-terminal residues would result in low affinity interaction with IMPα/β and accompanying lower efficiency nuclear import, to enable UL44 to interact with several proteins before being translocated into the nucleus. These include the catalytic subunit of the DNA polymerase holoenzyme, UL54 [54], the uracil DNA glycosylase UL114 [104] and UL44 itself [105,106]. Thus, it seems likely that the essential role of the UL44 *C*-terminus in HCMV replication depends on its nuclear transport regulation properties, and the regulation thereof by phosphorylation in particular. Of importance in this context is firstly that all nuclear transport enhancing sites (S413, S415 and S418) have been found to be phosphorylated to high levels in cells infected with HCMV *in vitro* [103], while the introduction of phosphonull mutations at these sites abolishes both UL44 nuclear accumulation and viral replication [103]. Secondly, introduction of phosphonull or phosphomimetic mutations on T427 decreases HCMV DNA replication in *OriLyt* transcomplementation assays [69]. Thirdly, UL44 is known to transport to the nucleus other viral factors, including the uracil-DNA glycosylase UL114 [104] and the UL54 pol catalytic subunit [54], so that regulation of its nuclear transport activity would also influence the subcellular localization of these factors (Figure 3). Finally, the key phosphorylation sites are all conserved in other β-Herpesviruses, including HHV-6A and 6B and HHV-7 [52].

However, it is also possible that the role of the UL44 *C*-terminus in HCMV replication does not relate exclusively to its ability to confer phosphorylation-dependent nuclear accumulation, since the HCMV *C*-terminus is capable of conferring binding to factors other than BRAP2 and IMPs, including the viral phosphoprotein UL112-113 p84 [96] and the SUMO E2 conjugase Ubc9 [97]; the exact role these interactions play in the HCMV life cycle, however, is unclear. What is clear is that phosphorylation through a CK2-triggered phosphorylation cascade [69] of UL44 residues S413, S415 and S418 is absolutely required for nuclear targeting and viral replication [103], implying that pharmacological inhibition of CK2 activity would also result in inhibition of viral replication. Whether recently developed, highly selective CK2 inhibitors, [107] may have application in anti-HCMV therapy remains to be seen, and cell toxicity associated to the use of such compounds will have to be considered [108].

## 7. Nuclear Import of DNA Primase Helicase Complexes and the Formation of Composite NLSs

Despite the fact that several subunits of the polymerase holoenzyme possess functional NLSs, such signals have still not been identified on any herpesviral primase/helicase subunit. Indeed, ectopic expression of any of these three subunits in isolation has always been reported to be cytoplasmic [61,63,64,109], despite the fact that all three clearly need to be translocated to the nucleus in order to perform their role in viral replication, and can be readily detected in the nucleus of infected cells [109,110]. In all studies to date, the simultaneous expression of at least all three subunits appears to be necessary for nuclear targeting; in the case of HSV-1 and EBV, coexpression of the three subunits appears to be sufficient for nuclear targeting of the complexes [61,63,64]. In the case of KSHV, the DNA polymerase holoenzyme and the ssDNA binding protein also need to be coexpressed with the helicase/primase holoenzyme to ensure nuclear localization [62]. Thus, herpesviruses appear to have evolved mechanisms to prevent nuclear entry of individual, unassembled helicase/primase subunits, presumably to avoid their potential interaction with cellular rather than viral DNA [61,62,63,64]. 

Based on these observations, it is currently believed that assembly of the DNA helicase/primase holoenzymes generates an as yet unidentified but functional NLS, which may depend on conformational changes induced by interaction of the subunits either to unmask an otherwise masked nuclear targeting peptide, or to create a composite NLS which is formed by amino acids belonging to each individual subunits. Consistent with the first possibility, bioinformatics analysis has identified putative hPY-NLSs on HSV-1 PAF UL8 and KSHV primase ORF52 (respectively PGALAPVFAFLGPEFEVRGGPVPY_358_ and TFQSQVAWLRTKFVTALRKLYKMTPSPY_160_), although it remains to be elucidated whether these signals are functional. In the case of KSHV, this scenario is completed by the evidence that PAF (ORF40/41) can also be partially translocated to the nucleus when expressed in the presence of other viral proteins, such as K8 or MTA, implying that piggy-back transport through individual viral factors may also contribute to nuclear accumulation [62]. The IMPs responsible have not been identified, but a recent report has implicated the cellular chaperone DNAJB6 in the nuclear transport of HCMV primase (UL70) [109]. Significantly, DNAB6 is not the only cellular chaperone playing a role in the nuclear targeting of herpesviral replicating proteins (see below).

## 8. An Emerging Role for Cellular Chaperones in Nuclear Import

There is growing evidence that chaperones such as heat shock protein (Hsp) 90 and Hsp40 play an important role in nuclear targeting of herpesviral DNA replication proteins. The first example is represented by HSV-1 UL30, which bears two independently acting NLSs (see Table 2 and Figure 2) and which can localize to the nucleus independently of its cognate PAP [53,55,56]. Once inside the cell nucleus, UL30 co-localizes in replication compartments with Hsp90. Drug-mediated inhibition of Hsp90 activity resulted in cytoplasmic retention of UL30 and its degradation via the proteasome [87]. The inability of UL30 to reach the nuclear compartment is probably the reason for the simultaneous drop in viral DNA replication and infectious particle production observed upon inhibition of Hsp90 in HSV-1 infected cells *in vitro* [87]. It is likely that Hsp90 activity is required for correct folding of UL30, thus exposing the NLSs and promoting their interaction with IMPs for nuclear targeting. Importantly, a recent report showed that either pharmacological or genetic ablation of Hsp90 causes a drop in EBV DNA replication during lytic infection [60]. As in the case of HSV-1, this inhibition is most likely dependent on the observed mislocalization of the EBV pol (BALF1) to the cytosol. Immunoprecipitation data suggested that Hsp90 is required to enable BALF1 to interact with BMRF1. Therefore, while in the case of HSV-1, Hsp90 activity is likely required for correct folding of UL30 to enable interaction with IMPs/subsequent nuclear transport, Hsp90 probably assists the folding of EBV BALF1 to enable its interaction with BMRF1, which then drives nuclear targeting of the DNA polymerase holoenzyme through its *C*-terminally located cNLS (see Table 2 and Figure 2) [58]. Another herpesvirus whose life cycle can be targeted by Hsp90 inhibitors is VZV, where nuclear localization of the VZV ssDNA-binding protein ORF29p relies on Hsp90 activity. ORF29p localizes to the nucleus of infected cells during lytic infection, and when individually expressed in the absence of other viral proteins, through an IMPα/β recognized, *N*-terminally located atypical NLS of 144 aas [111]; inhibition of Hsp90 activity by geldanamycin results in ORF29p mislocalization to the cytoplasm, where it is rapidly subjected to proteasomal degradation [112]. Moreover in the case of VZV-infected cells, Hsp90 inhibition causes a dramatic reduction of both viral genome replication and viral progeny production. Interestingly, although colocalizing with Hsp90, ORF29 does not appear to bind Hsp90 directly, but potentially indirectly through the host cochaperone/adaptor protein BAG3 [112]. Consistent with this idea, siRNA-mediated inhibition of BAG3 expression results in delayed/ineffective VZV spread and replication [112].

Taken together these data indicated that various herpesviruses can exploit the activity of Hsp90 in order to enable viral lytic cycle progression, implying that Hsp90 may represent a common target for approaches to prevent herpesvirus replication. Indeed pharmacological ablation of HSP90 inhibits the HSV-1, EBV and VSZ life cycles [60,87,112]. Consistent with the idea that chaperone proteins may play a role in infection by all herpesviruses is the fact that HCMV primase UL70 has recently been shown to interact with the Hsp40 family member DNAJB6. Two different isoforms of DNAJB6 protein, generated by alternative splicing, have been thus far identified: DNAJB6-a and DNAJB6-b, which exhibit distinct subcellular localization. While DNAJB6-a is nuclear, DNAJB6-b localizes exclusively in the cytoplasm, possibly through the fact that DNAJB6-b lacks the *C*-terminal domain, which contains a functional NLS [113]. Both isoforms interact with UL70, as demonstrated in the yeast two hybrid system and coimmunoprecipitations, suggesting that the interaction does not require the *C*-terminal domain of DNAJB6 [109]. The relative levels of DNAJB6 isoforms appear to play a crucial role in regulating the subcellular localization of HCMV UL70 protein. As reported for several herpesviral helicase/primase subunits, individual expression of UL70 in the absence of other viral proteins results in cytoplasmic localization [109]. Upon coexpression with DNAJB6-a, but not DNAJB6-b, UL70 accumulates strongly in the nucleus, suggesting that DNAJB6-a plays a crucial role in mediating UL70 nuclear accumulation, presumably via a piggy-back mechanism. In contrast, cells overexpressing HCMV primase and DNAJB6-b showed marked cytoplasmic localization of both proteins. Similar results have been obtained in HCMV-infected cells, where siRNA-mediated knockdown of DNAJB6-a or overexpression of DNAJB6-b prior to infection resulted in dramatically reduced HCMV replication and virus production, while silencing of DNAJB6-b or overexpression of DNAJB6-a causes an increase in HCMV replication and virus production [109]; levels of HCMV replication and virus production correlate well with nuclear accumulation of myc-tagged cotransfected UL70. Thus, it appears that HCMV has evolved mechanisms to exploit variations in the levels of DNAJB6 isoforms to regulate the subcellular localization of its primase UL70 and possibly the whole DNA primase/helicase complex. 

## 9. Conclusions

Nuclear targeting of herpesvirus DNA replication factors remains largely uncharacterized, despite the fact that it represents a potential target for therapeutic intervention. Key unanswered questions include why HSV-1 and HCMV DNA polymerase holoenzymes subunits can be imported as individual proteins or in a complex, while this is not the case for γ-herpesviruses [52,53,54,55,56,59,60]; why HCMV evolved such a sophisticated mechanism of regulation by multiple phosphorylation sites of nuclear targeting of its PAP [51,52,69,95], and whether Hsp90 activity is required for other herpesviral proteins in addition to EBV BALF5, HSV-1 UL30 and VZV protein ORF29p [60,87,112]. In addition, the exact mechanism enabling nuclear transport of helicase/primase complexes, as well as the cellular factors (including IMPs) responsible are still unknown [61,62,63,64,109]. 

Answering these questions is not only important for fundamental understanding of herpesviral biology, but also for the development of urgently needed antiviral drugs to fight herpesvirus infections. Inhibition of IMPα/β mediated nuclear import has been recently shown to impair both HIV-1 and DENV replication [89,90]; since several crucial herpesviral proteins are dependent on IMPα/β for transport to the nucleus, it seems highly likely that pharmacological targeting of IMPα/β may impact on herpesvirus production. Along the same lines, it is possible that inhibition of kinases phosphorylating the *C*-terminus of HCMV PAP, including CK2, may result in impaired HCMV replication, by impairing PAP nuclear import. Finally, Hsp90 inhibitors may represent a novel class of anti-herpetic drugs able to block nuclear import of several DNA replicating machinery proteins. Further studies will be required to verify all of these exciting possibilities. 
